# *In vitro* analysis of phosphorothioate modification of DNA reveals substrate recognition by a multiprotein complex

**DOI:** 10.1038/srep12513

**Published:** 2015-07-27

**Authors:** Bo Cao, Xiaoqing Zheng, Qiuxiang Cheng, Fen Yao, Tao Zheng, I. Ramesh Babu, Huchen Zhou, Peter Dedon, Delin You

**Affiliations:** 1 State Key Laboratory of Microbial Metabolism, and School of Life Sciences & Biotechnology, Shanghai Jiao Tong University, Shanghai, China 200030; 2 Department of Biological Engineering, Massachusetts Institute of Technology, 77 Massachusetts Avenue, Cambridge, MA 02139; 3 Singapore-MIT Alliance for Research and Technology, 1 CREATE Way, Singapore 138602; 4 Joint International Research Laboratory of Metabolic and Developmental Sciences, Shanghai Jiao Tong University, Shanghai, China 200240

## Abstract

A wide variety of prokaryotes possess DNA modifications consisting of sequence-specific phosphorothioates (PT) inserted by members of a five-gene cluster. Recent genome mapping studies revealed two unusual features of PT modifications: short consensus sequences and partial modification of a specific genomic site in a population of bacteria. To better understand the mechanism of target selection of PT modifications that underlies these features, we characterized the substrate recognition of the PT-modifying enzymes termed DptC, D and E in a cell extract system from *Salmonella*. The results revealed that double-stranded oligodeoxynucleotides underwent *de novo* PT modification *in vitro*, with the same modification pattern as *in vivo*, *i. e.*, G_ps_AAC/G_ps_TTC motif. Unexpectedly, in these *in vitro* analyses we observed no significant effect on PT modification by sequences flanking GAAC/GTTC motif, while PT also occurred in the GAAC/GTTC motif that could not be modified *in vivo*. Hemi-PT DNA also served as substrate of the PT-modifying enzymes, but not single-stranded DNA. The PT-modifying enzymes were then found to function as a large protein complex, with all of three subunits in tetrameric conformations. This study provided the first demonstration of *in vitro* DNA PT modification by PT-modifying enzymes that function as a large protein complex.

Though known for several decades as a synthetic DNA modification^1^,^2^,^3^,^4^,^5^, sulfur modification of the DNA backbone in the form of a phosphorothioate (PT) was recently discovered in a wide variety of taxonomically unrelated bacteria^6^,^7^,^8^,^9^. A family of five-gene clusters was found to be responsible for the incorporation of sulfur into the DNA backbone to form PT modifications in a sequence- and stereo-specific manner^8^. In *Salmonella enterica* serovar Cerro 87, PT modifications function in a host restriction-modification system, with restriction provided by genes *dptF, G* and *H* that cluster with modification genes *dptB-E*^9^,^10^,^11^. However, the function of PT modifications in organisms lacking *dptF-H*, such as *Vibrio cyclitrophicus* FF75^9^, is not known. Further, the lack of *in vitro* methods for studying PT biochemistry has limited our understanding of the molecular mechanisms of PT insertion and restriction, and the mechanism of genomic target selection by PT-modifying proteins.

Our current understanding of the biosynthesis of PT modifications involves a poorly defined but complex interaction of proteins coded by the five PT-modifying genes, designated as *dnd* or *dpt* A-E^6^,^10^. For example, while an endogenous cysteine desulfurase gene *iscS* often replaces *dndA*^10^,^12^, DndA of *S. lividans* 66 acts as a cysteine desulfurase and assembles DndC as a 4Fe-4S cluster protein^13^, while DndC possesses ATP pyrophosphatase activity and is predicted to have PAPS reductase activity^13^. DndB has homology to a group of transcriptional regulators and its absence leads to increased level of PT modifications on genome^20^. A DndD homologue in *Pseudomonas fluorescens* Pf0-1, SpfD, has ATPase activity and is possibly involved in DNA structure alteration or nicking during PT modification^14^. DndE structure reveals a tetrameric conformation with nicked DNA binding activity^15^. However, the mechanisms of substrate recognition and the coordination of biochemical steps in PT synthesis are not known.

Further complicating our understanding of PT biology is the recent observation in *Escherichia coli* B7A and *Vibrio cyclitrophicus* FF75 that only a small fraction of unusually short consensus sequences are modified with PT^9^. PT modifications occur on both DNA strands at G_ps_AAC/G_ps_TTC motifs in *E. coli* B7A genome while *V. cyclitrophicus* FF75 presents DNA single-strand modifications with a C_ps_CA pattern, with no further strict consensus sequences beyond the modification motifs in both cases^9^. More importantly, only 12% of the GAAC/GTTC sites are modified in *E. coli* B7A in spite of the presence of the PT-dependent restriction system in this strain^9^. This partial PT modification phenomenon was also observed in *V. cyclitrophicus* FF75, which lacks the PT restriction system, with 14% of possible CCA sites modified^9^. These observations raise questions about DNA substrate recognition and selection by PT-modifying enzymes. To address these problems, we used an *in vitro* PT modification system and affinity purification techniques to analyze the interactions and target recognition properties of PT-modifying proteins.

## Results and Discussion

### *In vitro* and *de novo* PT modification of double-stranded oligonucleotides

**Figure 1:**
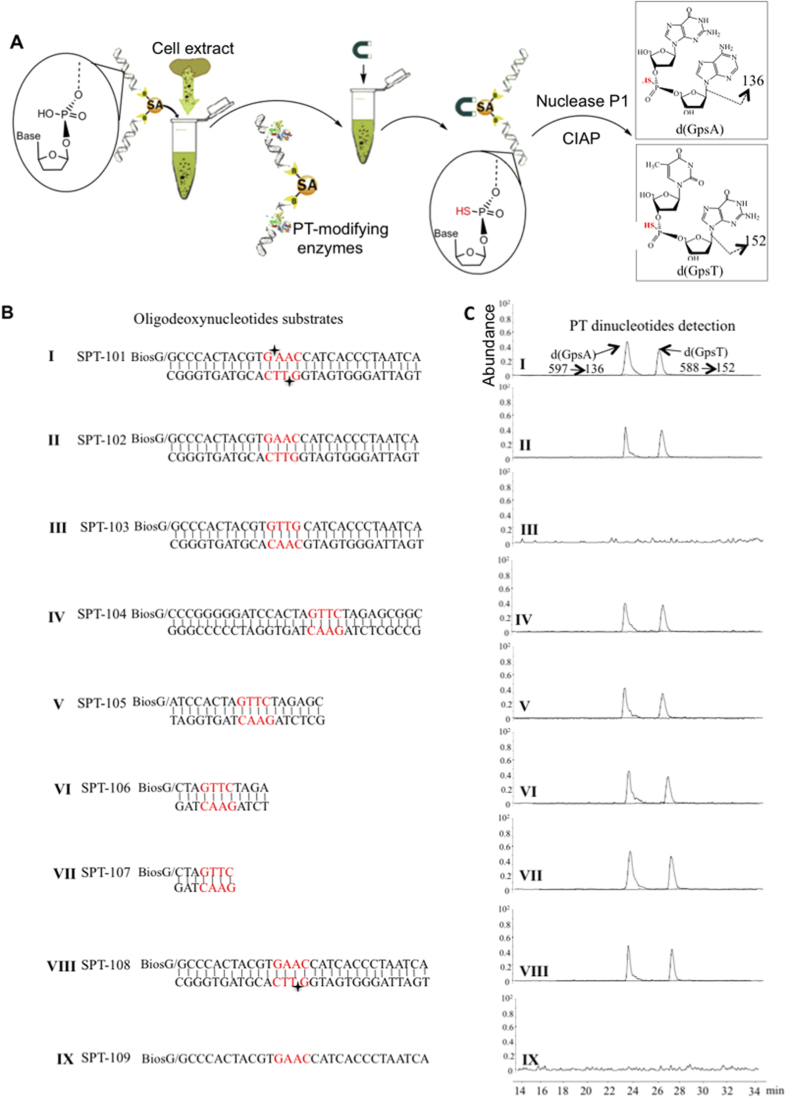
*De novo* PT modification in *S. enterica* serovar Cerro 87 cell extract system. (**A**) The schematic illustration of the strategy for *in vitro* PT modification by the cell extract system (SA, Streptoavidin; CIAP, calf intestinal alkaline phosphatase). (**B**) The sequences of the substrates are listed, with “♦” indicating the phosphorothioate position in the GAAC/GTTC sites. (**C**) Q-TOF mass spectrometer analysis of the PT dinucleotides G_ps_A (precursor ion m/z, 597; product ion m/z, 136) and G_ps_T (precursor ion m/z, 588; product ion m/z, 152) that was modified by cell extract system with different substrates in Fig. 1B. Figure was drawn by Bo Cao.

To better understand the biochemistry of PT-modifying enzymes, we refined and extended a rudimentary *in vitro* cell extract PT modification system^9^ and applied it to extracts from *S. enterica* serovar Cerro 87, which contains PT-modifying genes *dptB-E* and a cysteine desulfurase gene *iscS* in place of *dptA*^9^,^10^. In this cell extract assay system, biotinylated oligodeoxynucleotides are bound to streptavidin-coated agarose beads and then mixed with buffered cell-free extract, ATP, L-cysteine and pyridoxal phosphate to initiate the phosphorothioation reaction^9^, as illustrated in Fig. 1A. Following washing steps, the oligos are enzymatically hydrolyzed to release 2-deoxynucleosides and PT-linked dinucleotides, with the latter monitored by chromatography-coupled tandem mass spectrometry^9^. Here, we used this system to assess the substrate properties of the PT-modifying proteins DptB, C, D, and E in cell-free extracts of *S. enterica* serovar Cerro 87, in which IscS protein replaces DndA as the cysteine desulfurase^6^,^10^,^12^. The PT modification pattern in this strain was previously observed to occur as bistranded modifications at G_ps_AAC/G_ps_TTC motifs with no wider consensus sequence^9^. The first series of 29 bp duplex oligos, SPT-101, -102, and -103 (Fig. 1B), represent a duplex sequence context observed in genome mapping studies to be among the PT modified GAAC/GTTC sites^9^. SPT-101 contains synthetic PT modifications and was used to validate the analytical method applied to the bead-based affinity purification system (Fig. 1B). As shown in Fig. 1C, the generation of d(G_ps_A) and d(G_ps_T) in SPT-102 establishes *de novo* synthesis of PT by PT-modifying enzymes in *S. enterica* serovar Cerro 87 extract, which is consistent with the *in vivo* modification pattern as G_ps_AAC/G_ps_TTC^9^. As expected, PT was not incorporated in SPT-103 in which the GAAC/GTTC consensus motif was scrambled (GTTG/CAAC) (Fig. 1B). Unexpectedly, the substrate SPT-104, in which the GAAC/GTTC motif was situated in a sequence context that was not modified *in vivo*, served as an efficient substrate in the cell extract modification system (Fig. 1B). This suggests a wider substrate specificity for PT-modifying enzymes with oligodeoxynucleotides substrates *in vitro* than with genomic DNA *in vivo*, which is consistent with a target selection mechanism that uses cues that lie outside the 29 bp boundary of the GAAC/GTTC motif to fine-tune binding to the motif.

### Sequences flanking the GAAC/GTTC motif have little effect on PT modification *in vitro*

The previous genome mapping studies established the absolute requirement for the GAAC/GTTC motif in substrate recognition by PT-modifying proteins. To assess the role of flanking sequences in PT modification, we examined substrates with GAAC/GTTC motifs that, except for their length, had sequence contexts identical to SPT-103 (SPT105-107). As revealed in the *in vitro* assay results in Fig. 1C, all of these substrates were modified with PT at the GAAC/GTTC motif. Notably, even the 7 bp substrate with a 3′-terminal GAAC/GTTC motif could be modified. These results indicate that the only requirement for PT modification *in vitro* is the 4 bp GAAC/GTTC motif, with no significant effect by flanking sequences.

### Hemi-modified oligos are substrates for further PT modification

The post-replicative PT modification participates in a restriction-modification system in *S. enterica* serovar Cerro 87^10^, which suggested that genomic PT patterns would be clonally inherited in a semi-conservative manner, with PT-modifying enzymes catalyzing PT incorporation opposite PT-modified sites at GAAC/GTTC consensus sequences. To test this hypothesis, we used a version of the native modification context on SPT-101 and -102 in which PT was present on only on strand (SPT-108). As shown in Fig. 1C, PT was incorporated into this hemi-modified substrate to produce the fully modified, bistranded product. However, PT-modifying enzymes did not react with the unmodified strand when used as single-stranded oligodeoxynucleotide (SPT109, Fig. 1B), which is consistent with the observation that DndE, a DNA binding protein during PT modification, showed no single-stranded DNA binding activity^15^.

### DptC, D and E function as a large protein complex

**Figure 2:**
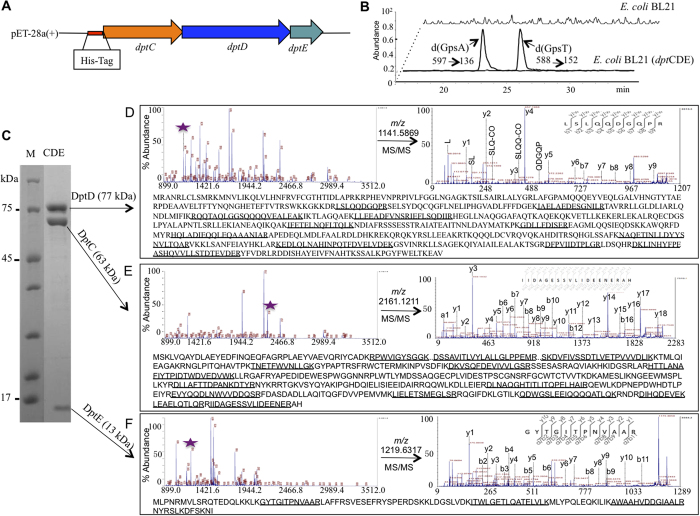
Identification of DptC/D/E enzymes functioning as a protein complex. (**A**) Heterologous co-overexpression of *dptCDE* operon by labeling DptC with His-tag. (**B**) Detection of PT modification in the host strain of *E. coli* BL21 harboring heterologous *dptCDE* gene cluster. (**C**) SDS-PAGE analysis of the proteins co-purified with DptC. (**D**–**F**) Mass spectra of DptD, DptC, DptE, respectively, digested with trypsin by MALDI-TOF/TOF MS (left panels) and an analysis of MS/MS spectrum [M + H]^+^ acquired for the tryptic peptides with sequences listed (right panels); All the detected peptides by MALDI-TOF-TOF are underlined at each bottom.

Further insight into PT-modifying protein function was gained using a pull-down assay to investigate the interactions of proteins DptC-E. In this assay, DptC was labelled with an N-terminal histidine-tag and then heterologously co-overexpressed with PT-modifying proteins D and E in *E. coli* BL21 (Fig. 2A). That these *S. enterica* serovar Cerro 87 proteins perform PT modification with the endogenous *E. coli* BL21 IscS protein^12^ was verified by LC-MS/MS analysis of d(G_ps_A) and d(G_ps_T) in genomic DNA isolated from bacteria transformed with the expression vector (Fig. 2B); non-transformed bacteria lacked the PT modifications. The labelled DptC was exploited as bait in pull-down studies using nickel affinity chromatography to purify DptC and any “pray” proteins, with analysis of the proteins by SDS-PAGE. As show in Fig. 2C, the most abundant proteins co-purifying with DptC were two proteins with masses similar to those of DptD (77 kDa) and DptE (13 kDa). The identities of the three proteins were then established by in-gel trypsin digestion and analysis of peptides by MALDI-TOF/TOF mass spectrometry. The mass spectrometric data were in good agreement with the predicted tryptic peptide mass fingerprinting of DptC, D and E (Fig. 2D–F), suggesting that PT-modifying proteins had strong interactions and functioned as a protein complex. Although the *E. coli* IscS has been demonstrated to participate in PT modification as a cysteine desulfurase^12^, it was not detected in the DptC pull-down studies, which suggests that it does not interact strongly with the DptC/D/E complex.

**Figure 3:**
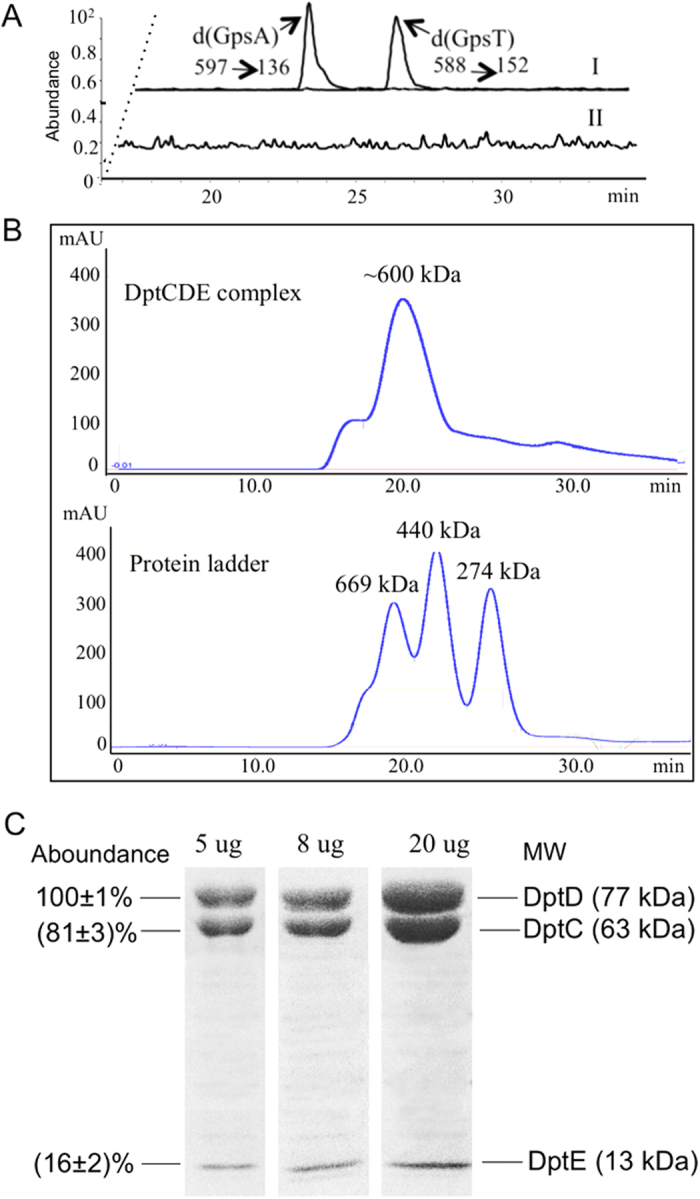
PT-modifying activity and characterization of purified DptC/D/E complex. (**A**) The purified DptC/D/E complex presented PT-modifying activity (I) in the cell extract of *S. enterica* serovar Cerro 87 mutant lacking DptC/D/E proteins (II). (**B**) Gel filtration chromatography analysis of DptC/D/E complex revealed a ~600 kDa molecular mass. (**C**) Quantification of the subunits of DptC/D/E complex on SDS-PAGE by ImageJ software. The relative amount of DptC and E were shown on the left by assuming DptD as 100%. Protein molecular sizes were listed on the right.

The PT-modifying activity of the purified DptC/D/E complex was then verified in the cell extract system of a *S. enterica* serovar Cerro 87 mutant lacking PT-modifying genes. As shown in Fig. 3A, the DNA substrate SPT-102 was modified by the purified DptC/D/E complex that was added to cell extracts lacking PT-modifying proteins, with modification occurring at the GAAC/GTTC consensus sequence. This result is consistent with DptC, D and E performing their function of PT incorporation as a protein complex. Furthermore, though *dptB* is located in the same operon with *dptCDE*, the DptB protein was not required for biosynthesis of PT modifications, which was consistent with that DptB functions as a transcriptional regulator of PT-modifying genes^6^,^7^,^8^.

The affinity-purified DptC, D and E protein complex was further characterized by gel filtration chromatography, which revealed that DptC, D and E co-chromatographed as a single peak corresponding to a molecular mass of ~600 kDa (Fig. 3B). The relative amount of each PT-modifying protein in the complex was determined by SDS-PAGE resolution of the co-eluting proteins in the 600 kDa fraction, with densitometry of individual bands (Fig. 3C). After normalization of the densitometry signal by the molecular sizes of DptC, D and E, the proteins were found to be present at equimolar amounts in the co-eluting protein complex. Considering its ~600 kDa molecular mass, the DptC/D/E complex appears to be comprised of four copies of each protein, which is consistent with the tetrameric structure of DptE in its crystal structure^15^.

Modification of DNA by PT-modifying protein families has emerged as a widespread feature of prokaryote physiology^6^,^7^,^8^, yet little is known about the mechanisms governing DNA target selection or the *in vivo* biochemistry of PT modification proteins. A recent study revealed that PT modifications occur at relatively small consensus sequences and that only a small fraction of possible these motifs are modified^9^, in some cases in spite of the presence of PT-dependent restriction enzymes^9^,^10^,^11^. This suggests an unusual target selection mechanism of PT-modifying enzymes. Our *in vitro* studies have now revealed that the PT-modifying proteins and IscS in *S. enterica* serovar Cerro 87 specifically modify both strands of GAAC/GTTC motifs, which is consistent with *in vivo* studies^9^. However, the observation that the PT-modifying/IscS proteins recognized GAAC/GTTC sites in 29 bp sequence contexts not modified *in vivo* (SPT104 *versus* SPT102) and that modification occurred in GAAC/GTTC sequences as short as 7 bp suggests that there are higher-order determinants for PT modification in the bacterial genomes. One possible explanation for the differences between *in vitro* and *in vitro* substrate specificity relates to the concentration of PT-modifying proteins *in vivo*, with expression of PT-modifying genes strictly regulated in cells and the relatively high concentration of PT-modifying proteins in our *in vitro* studies forcing modification at sub-optimal sites. This hypothesis is consistent with the previous observation that overexpression of PT-modifying genes increases the level of PT modifications with the same sequence motifs^7^. Another possibility is that PT modification enzymes *in vivo* have a preference for GAAC/GTTC motifs that possess a unique DNA secondary structure or shape determined by sequences longer than the 29 bp oligos used in the present studies. This is similar to the behavior enzymes such as DNA helicase BLM and WRN^16^, endonuclease FEN-1^17^ and the DNA nuclease Rad50/Mre11 complex^18^. Analysis of the purified DptC/D/E protein complex also provided important insights into PT-modifying target selection. In the purified complex, there were four copies of each protein, which is numerically consistent with the tetrameric conformer of the DptE protein in its crystal structure^15^. The biological relevance of this type of oligomeric protein complex is supported in numerous studies of other DNA modifying and restriction-modification systems, such as the heteropentameric structure of the Type I restriction-modification complexes^19^.

In summary, this study provided the first demonstration of *in vitro* DNA PT modification and revealed novel features of PT-modifying enzymes as a multiprotein complex. The selective modification of GAAC/GTTC sites points to sequence-specific modification as a critical feature of PT-modifying proteins, while the unselective modification of GAAC/GTTC sites in different sequence contexts not modified *in vivo* points to longer-range determinants of target selection by the PT-modifying proteins. Given the diversity of modification sequences by PT-modifying protein families across prokaryotic taxa, elucidation of these higher-order and longer-range influences will be critical to fully understanding the biology of PT modifications.

## Methods

### Materials, bacterial strains and culture conditions

All the oligodeoxynucetides used in this study were listed in Fig. 1B. Synthetic oligonucleotides were obtained from Shanghai Sangon Biotech (Shanghai). *S. enterica* serovar Cerro 87 wild-type strain and its derivative mutants were described in our previous study^10^. All the strains were cultured in Luria-Bertani (LB) medium at 37 °C to exponential growth phase (OD_600_ = 1.0) and then used for cell-free extract preparation.

### Cell extract system assay of DNA phosphorothioate modification

The *in vitro* cell extract assay was based on previous studies^9^. The detection of PT dinucleotides was accomplished by LC-MS/MS analysis using an Agilent SB-C18 column (150 × 2.1 mm, 3.5 μm) with a flow rate of 0.3 mL/min and the following parameters: column temperature: 35 °C; solvent A: 0.1% acetic acid; solvent B: 0.1% acetic acid in acetonitrile; gradient: 3% B for 5 min, 3% to 15% B over 20 min, and 15% to 100% B over 1 min. The HPLC column was coupled to an Agilent 6410 Q-TOF mass spectrometer with an electrospray ionization source in positive mode with the following parameters: gas flow, 10 L/min; nebulizer pressure, 30 psi; drying gas temperature, 325 °C; and capillary voltage, 3,100 V. Multiple reaction monitoring mode was used for detection of product ions derived from the precursor ions, with all instrument parameters optimized for maximal sensitivity (retention time in min, precursor ion *m/z*, product ion *m/z*, fragmentor voltage, collision energy): d(G_ps_A), 20.5, 597, 136, 120 V, 40 V; d(G_ps_T), 26.5, 588, 152, 110 V, 17 V.

### Heterologous expression and purification of PT-modifying protein complex

The coding region of *dptC/D/E* operon [Genbank accession number GQ863484]^10^ was amplified via PCR using *S. enterica* serovar Cerro 87 genomic DNA as the template. The following oligonucleotide primers were used: upstream primer, 5′-TTGCCATATGAGTAAATTAGTTCAGGC-3′, NheI underlined; downstream primer, 5′- CGCGGATCCTATGGCACCGTTCATGGTGC3′, BamHI underlined. The ~4 kb PCR product containing *dptCDE* was amplified by DNA polymerase KOD-Plus (TOYOBO, Japan), with 30 seconds at 60 °C for annealing and 6 minutes at 68 °C for extension. The PCR product was then digested by NheI/BamHI and inserted into the corresponding sites of the pET-28a(+) expression vector (Novagen). The constructed plasmid was validated by DNA sequencing and then transformed into *E. coli* BL21 (DE3) to express DptC/D/E proteins with an N-terminal His-Tag in DptC. *E. coli* BL21 (DE3) harboring *dptC/D/E* was grown in LB medium to an OD_600_ of 0.8 at 37 °C, and subsequently induced at 30 °C for 8 h with 0.5 mM IPTG. The cultured cells were harvested by centrifugation and re-suspended in buffer A (20 mM Tris–HCl pH 7.4, 150 mM NaCl). The re-suspended bacteria were subjected to three complete freeze–thaw cycles and then sonically lysed. After centrifugation at 15,000 × g for 30 minutes, the supernatant was applied to a HiTrap chelating column charged with nickel (GE Healthcare). Elution was carried out with buffer B (20 mM Tris–HCl pH 7.4, 150 mM NaCl, 500 mM imidazole) from 0% to 60% over 20 min. Fractions containing His-Tag labeled DptC were pooled and collected. The proteins in the collected fractions were then determined according to their molecular weight by 12% SDS-PAGE with the protein marker (Protein Molecular Weight Marker, Beyotime, China).

### Identification of the protein complex of DptCDE

The purified His-Tag labeled DptC and the captured proteins were applied to Superdex Gel Filtration Column 10/300 GL (GE Healthcare) using buffer A (20 mM Tris–HCl pH 7.4, 150 mM NaCl) at a flow rate 0.2 mL/min. The protein marker (Gel Filtration Calibration Kit HMW, GE Healthcare) was used in the same condition to determine the size of DptC/D/E complex. The collected protein from Gel Filtration Column was then applied to a SDS-PAGE analysis with concentration of 12% polyacrylamide. For the MALDI-TOF/TOF analysis, the protein bands from the SDS-PAGE were individually subjected to in-gel trypsin digestion at 37 °C for 20 h. The samples were prepared for MALDI-TOF/TOF (4800 Plus MALDI TOF/TOF Analyzer, ABI) analysis after sonication by adding of 100 μL 60% ACN/0.1%TFA. MALDI-TOF/TOF analysis was performed in positive mode under the condition: matrix, CHCA (Sigma); laser power, 355 nm; accelerating voltage, 2 kV; MS range, 800–4000 Da. The MS and MS/MS data were then submitted to the Mascot database (http://www.matrixscience.com/cgi/search_form.pl?FORMVER=2&SEARCH=PMF) to analyze the peptides mass fingerprinting of the proteins.

### Quantification of subunits of DptCDE complex

Quantification of subunits of DptC/D/E complex was performed by ImageJ software (http://rsb.info.nih.gov/ij/). DptC/D/E complex collected from Superdex Gel Filtration Column was diluted to a series concentrations determined by Bioanalyzer 2100 (Agilent), and subjected to SDS-PAGE analysis. The protein bands on the Coomassie blue stained SDS-PAGE were then quantified by ImageJ software. The relative amounts of DptC, D and E were calculated after normalization by their molecular mass.

### Validation of the PT-modifying activity of DptC/D/E complex

The PT-modifying activity of the purified DptC/D/E complex was verified in the cell extract system of a *S. enterica* serovar Cerro 87 mutant XTG102^10^ that lacks the PT-modifying gene cluster (Δ*dptB-E*). The cells of XTG102 were collected by centrifugation at 3,000 × g for 3 minutes at its exponential growth phase (OD_600_ = 1.0) in LB medium at 37 °C. The cell extract was prepared according to our previous studies^9^. Dpt protein complex was added to the prepared cell extract with a concentration of 0.1 mg/mL and then the *in vitro* cell extract assay was performed based on previous study^9^.

## Additional Information

**How to cite this article**: Cao, B. *et al.* *In vitro* analysis of phosphorothioate modification of DNA reveals substrate recognition by a multiprotein complex. *Sci. Rep.* **5**, 12513; doi: 10.1038/srep12513 (2015).

## References

[b1] Verma S. & Eckstein F. Modified oligonucleotides: synthesis and strategy for users. Annu Rev Biochem 67, 99–134 (1998). 9759484 10.1146/annurev.biochem.67.1.99

[b2] Gish G. & Eckstein F. DNA and RNA sequence determination based on phosphorothioate chemistry. Science 240, 1520–1522 (1988). 2453926 10.1126/science.2453926

[b3] Stein C.A. Exploiting the potential of antisense: beyond phosphorothioate oligodeoxynucleotides. Chem Biol 3, 319–323 (1996). 8807859 10.1016/s1074-5521(96)90113-1

[b4] Matsukura M. *et al.* Phosphorothioate analogs of oligodeoxynucleotides: inhibitors of replication and cytopathic effects of human immunodeficiency virus. Proc Natl Acad Sci USA 84, 7706–7710 (1987). 3499613 10.1073/pnas.84.21.7706 PMC299369

[b5] Matsukura M. *et al.* Regulation of viral expression of human immunodeficiency virus *in vitro* by an antisense phosphorothioate oligodeoxynucleotide against rev (art/trs) in chronically infected cells. Proc Natl Acad Sci USA 86, 4244–4248 (1989). 2471199 10.1073/pnas.86.11.4244 PMC287427

[b6] Zhou X. *et al.* A novel DNA modification by sulphur. Mol Microbiol 57, 1428–1438 (2005). 16102010 10.1111/j.1365-2958.2005.04764.x

[b7] Wang L. *et al.* DNA phosphorothioation is widespread and quantized in bacterial genomes. Proc Natl Acad Sci USA 108, 2963–2968 (2011). 21285367 10.1073/pnas.1017261108 PMC3041111

[b8] Wang L. *et al.* Phosphorothioation of DNA in bacteria by *dnd* genes. Nat Chem Biol 3, 709–710 (2007). 17934475 10.1038/nchembio.2007.39

[b9] Cao B. *et al.* Genomic mapping of phosphorothioates reveals partial modification of short consensus sequences. Nat commun 5, 3951 (2014). 24899568 10.1038/ncomms4951 PMC4322921

[b10] Xu T., Yao F., Zhou X., Deng Z. & You D. A novel host-specific restriction system associated with DNA backbone S-modification in *Salmonella*. Nucleic Acids Res 38, 7133–7141 (2010). 20627870 10.1093/nar/gkq610 PMC2978375

[b11] Cao B. *et al.* Pathological phenotypes and *in vivo* DNA cleavage by unrestrained activity of a phosphorothioate-based restriction system in *Salmonella*. Mol Microbiol 93, 776–785 (2014). 25040300 10.1111/mmi.12692 PMC4414249

[b12] An X. *et al.* A novel target of IscS in *Escherichia coli*: participating in DNA phosphorothioation. PloS one 7, e51265 (2012). 23240007 10.1371/journal.pone.0051265 PMC3519819

[b13] You D., Wang L., Yao F., Zhou X. & Deng Z. A novel DNA modification by sulfur: DndA is a NifS-like cysteine desulfurase capable of assembling DndC as an iron-sulfur cluster protein in *Streptomyces lividans*. Biochemistry 46, 6126–6133 (2007). 17469805 10.1021/bi602615k

[b14] Yao F., Xu T., Zhou X., Deng Z. & You D. Functional analysis of *spfD* gene involved in DNA phosphorothioation in *Pseudomonas fluorescens* Pf0-1. FEBS lett 583, 729–733 (2009). 19171139 10.1016/j.febslet.2009.01.029

[b15] Hu W. *et al.* Structural insights into DndE from *Escherichia coli* B7A involved in DNA phosphorothioation modification. Cell Res 22, 1203–1206 (2012). 22525332 10.1038/cr.2012.66 PMC3391021

[b16] Mohaghegh P., Karow J.K., Brosh R.M. Jr., Bohr V.A. & Hickson I.D. The Bloom’s and Werner’s syndrome proteins are DNA structure-specific helicases. Nucleic Acids Res 29, 2843–2849 (2001). 11433031 10.1093/nar/29.13.2843 PMC55766

[b17] Harrington J.J. & Lieber M.R. The characterization of a mammalian DNA structure-specific endonuclease. EMBO J 13, 1235–1246 (1994). 8131753 10.1002/j.1460-2075.1994.tb06373.x PMC394933

[b18] Trujillo K.M. & Sung P. DNA structure-specific nuclease activities in the *Saccharomyces cerevisiae* Rad50*Mre11 complex. J Biol Chem 276, 35458–35464 (2001). 11454871 10.1074/jbc.M105482200

[b19] Kennaway C.K. *et al.* Structure and operation of the DNA-translocating type I DNA restriction enzymes. Genes & Development 26, 92–104 (2012). 22215814 10.1101/gad.179085.111 PMC3258970

[b20] Cheng Q. *et al.* Regulation of DNA phosphorothioate modifications by the transcriptional regulator DptB in *Salmonella*. Mol Microbiol doi: 10.1111/mmi.13096. 10.1111/mmi.13096 26096787

